# Alkaloid defenses of co-mimics in a putative Müllerian mimetic radiation

**DOI:** 10.1186/1471-2148-14-76

**Published:** 2014-04-04

**Authors:** Adam MM Stuckert, Ralph A Saporito, Pablo J Venegas, Kyle Summers

**Affiliations:** 1 Department of Biology, East Carolina University, 1000 E. Fifth St, Greenville, NC 27858, USA; 2 Department of Biology, John Carroll University, University Heights, Ohio 44118, USA; 3 División de Herpetología-Centro de Ornitología y Biodiversidad (CORBIDI), Santa Rita N°105 Of. 202, Urb. Huertos de San Antonio, Surco, Lima, Perú

**Keywords:** Alkaloids, Aposematism, Dendrobatids, Müllerian mimicry, Polytypism, *Ranitomeya imitator*

## Abstract

**Background:**

Polytypism in aposematic species is unlikely according to theory, but commonly seen in nature. *Ranitomeya imitator* is a poison frog species exhibiting polytypic mimicry of three congeneric model species (*R. fantastica, R. summersi,* and two morphs of *R. variabilis*) across four allopatric populations (a "mimetic radiation"). In order to investigate chemical defenses in this system, a key prediction of Müllerian mimicry, we analyzed the alkaloids of both models and mimics from four allopatric populations.

**Results:**

In this study we demonstrate distinct differences in alkaloid profiles between co-mimetic species within allopatric populations. We further demonstrate that *R. imitator* has a greater number of distinct alkaloid types than the model species and more total alkaloids in all but one population.

**Conclusions:**

Given that *R. imitator* is the more abundant species in these populations, *R. imitator* is likely driving the majority of predator-learned avoidance in these complexes. The success of *Ranitomeya imitator* as a putative advergent mimic may be a direct result of differences in alkaloid sequestration. Furthermore, we propose that automimicry within co-mimetic species is an important avenue of research.

## Background

Poison frogs provide a classic example of aposematism, in that they possess warning colors and/or patterns directed towards predators and are protected by alkaloid-based chemical defenses [1,2]. Alkaloid defenses have been detected in five families of poison frogs: Dendrobatidae [1,3], Mantellidae [4-6], Bufonidae in the genus *Melanophryniscus*[6-8], Myobatrachidae in the genus *Psuedophryne*[6], and recently in diminutive Cuban members of Eleutherodactylidae [9]. For a full review of the chemical sequestration in poison frog families see [10].

The family Dendrobatidae contains a high diversity of frog species and alkaloids [11,12], providing a number of unique opportunities to study the link between aposematism and chemical defense. Alkaloid defenses in all poison frogs (including Dendrobatidae) are sequestered from an arthropod diet [3], consisting primarily of mites, ants, beetles, and millipedes (reviewed in [13]). Accompanying the ability to sequester alkaloid defenses, many species exhibit substantial polytypism in color and pattern across their geographic range [14]. The presence of such polytypisms appears to contradict certain theoretical predictions, in particular, the hypothesis that predators exert strong selective pressure to maintain monomorphism as a result of learned avoidance [15]. Indeed, a number of studies using clay model replicas of dendrobatid frogs have shown that natural predators exert purifying selection, and “favor” one color morph within populations [16,17]; however, one study indicated that at low prey densities the effect of this stabilizing selection is limited [18]. In addition to phenotypic variation, many dendrobatids exhibit substantial variation in alkaloid defense, both geographically and temporally [1,3,19], and across and within populations [20].

**Figure 1:**
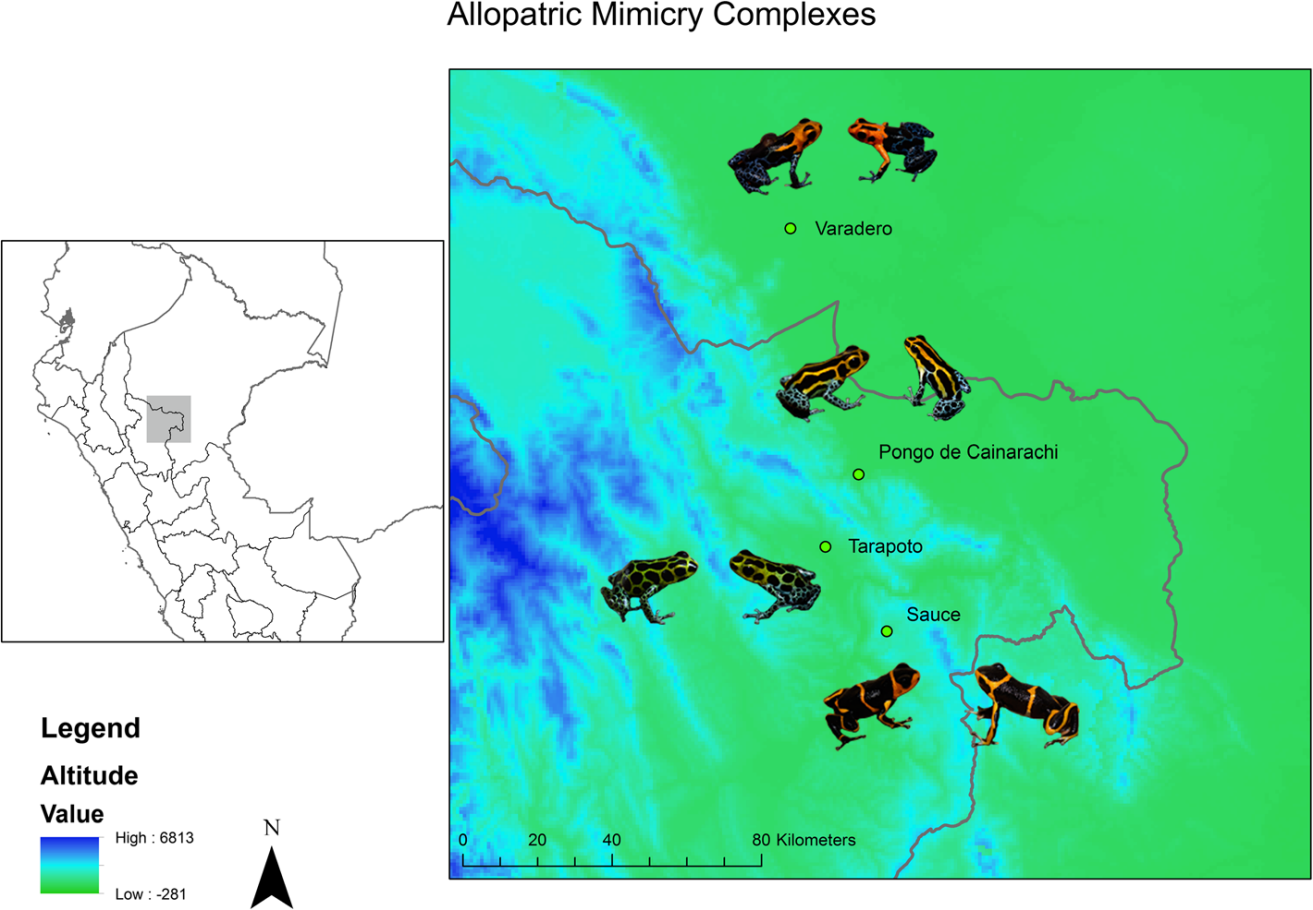
**Sampling species and localities for this study in the departments of San Martin and Loreto, Peru.** From North to South: *R. imitator* and *R. fantastica* from Varadero, Loreto; *R. imitator* and *R. variabilis* from Pongo de Cainarachi, San Martin; *R. imitator* and *R. variabilis* from Tarapoto, San Martin; and *R. imitator* and *R. summersi* from Sauce, San Martin. *Ranitomeya imitator* is the left species in each pairing, and the model species are on the right.

*Ranitomeya imitator*[21] is a polytypic poison frog species that appears to have gone through a rapid ‘mimetic radiation’ to adverge on to the morphological appearance of multiple congeneric species throughout its range [22-25]; however see [26]. Advergence is the process whereby one species evolves to appear similar to another (established) species, as opposed to convergence, a process in which two or more species evolve towards monomorphism [22,26]. There are four described allopatric mimetic populations of *R. imitator* in the departments of San Martin and Loreto in northern Peru (see Figure 1) [12,24]. *Ranitomeya imitator* was described as the advergent ‘mimic’ due to extremely short branch lengths in phylogenetic trees compared to its congeneric co-mimetic species *R. variabilis* (Zimmermann and Zimmermann) and *R. summersi*[12,22,23]. Chouteau *et al.*[26] raised concerns about the usage of these terms based on genetic differentiation between two close populations of co-mimetic *R. imitator* and *R. variabilis.* However, phylogeographic evidence provides additional support for the advergence hypothesis, with *R. imitator* mimicking two other (distantly related) species in other localities [12,22-24]. There is also evidence for recent divergence under selection in *R. imitator*[24], and evidence for multiple independent lineages of a highland spotted morph of *R. variabilis*, even in areas well outside the range of *R. imitator*[12]. Although we typically refer to these species as “co-mimics” throughout the paper, when relevant we use the term ‘mimic’ to refer to *R. imitator* and ‘model’ to refer to co-mimetic congeners (*R. fantastica, R. summersi,* and *R. variabilis*).

The mimetic complexes involving *Ranitomeya imitator* are considered an example of a Müllerian mimicry system in vertebrates [12,15,22,24], and provide a close parallel to the well-known Müllerian mimicry systems of *Heliconius* butterflies [27,28]. However, the hypothesis of Müllerian mimicry in *Ranitomeya* was only recently tested in a study describing reciprocal learned avoidance by predators between co-mimetic *R. variabilis* and *R. imitator*[29]*.* Reciprocal learned avoidance is a key tenet of Müllerian mimicry, and the results of Stuckert *et al.*[29] support the hypothesis of a Müllerian mimicry system—the first known in anurans. Other putative Müllerian complexes exist in anurans (e.g., mantellids [30], *Amereega picta* (Tschudi) and *Leptodactylus lineatus* (Schneider) [31], and among other members of the genus *Ranitomeya*[12]), but these have not been experimentally verified.

Another key tenet of the hypothesis of Müllerian mimicry is that co-mimetic species all possess a secondary defense (e.g., alkaloid defenses in *Ranitomeya*) against predators. Learned avoidance by predators in this system is seemingly a good indication of the presence of an alkaloid defense [29]. However, here, we explicitly test each mimetic species for the presence, quantity, and identity of alkaloids. The presence of alkaloids in these species would provide significant support for the hypothesis that this is a Müllerian mimicry system.

Because alkaloid defenses in poison frogs are sequestered from dietary sources [3,13], and the species we are examining are congenerics with similar ecologies, we would expect sympatric Müllerian co-mimics in this system to possess similar defense profiles. Alternatively, mimetic species that differ in their chemical protection may not behave as Müllerian mimics, even if they are both chemically defended. Instead, differences in defensive chemicals between species may decrease the efficiency of learned avoidance, in particular when predators sample prey that are less toxic (i.e., palatable) than individuals sampled previously [15,32]; however see [33] for counter-arguments. In the present study, we further examine the hypothesis that the co-mimetic species are Müllerian mimicry systems by examining alkaloid defenses between co-mimetic species and broadening the scope of examined mimetic complexes.

To investigate the relationships between chemical defenses among co-mimetic species, we characterized the alkaloids of *R. imitator* and its congeneric co-mimics (*R. variabilis, R. fantastica,* and *R. summersi*) in four allopatric mimetic complexes. This study covers the majority of the range of *R. imitator* and examines all known mimetic complexes of the species. In this paper, we present a detailed study of the chemical secondary defenses in the genus *Ranitomeya,* as well as the only study of chemical secondary defenses in the context of Müllerian mimicry in amphibians. Our study provides valuable insight into the workings of the only confirmed system of Müllerian mimicry in anurans. It thus has substantial implications for our understanding of mimicry in other mimetic anuran systems, for parallel systems in *Heliconius* butterflies, and for Müllerian mimicry in general.

## Results

**Figure 2:**
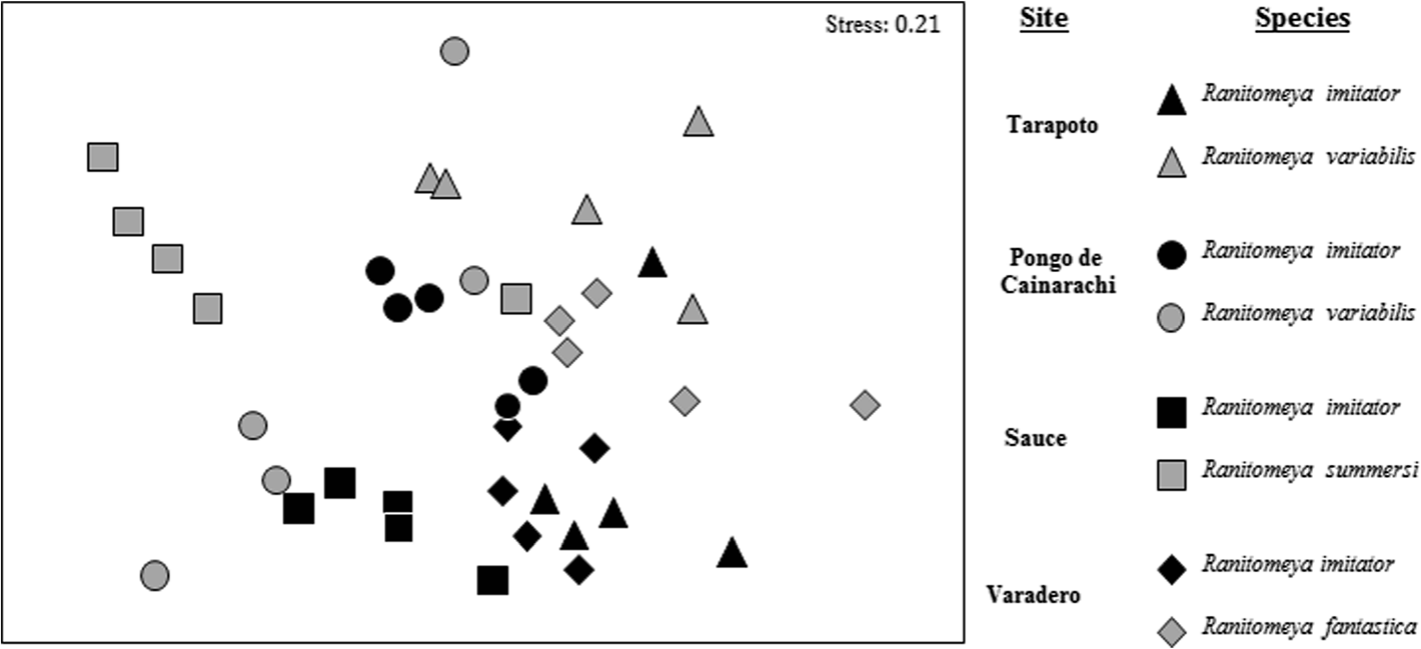
**nMDS plot of alkaloid composition among individual frogs and species from different sites.** Each symbol represents an individual frog and species from a specific site, and the distance between symbols represents the difference in alkaloid composition. Alkaloid composition varies significantly among sites (Global R = 0.331; p = 0.001), and between species within each site (p < 0.05 for all within site comparisons).

Alkaloid composition varied significantly among sites (Global R = 0.331; p = 0.001; Figure 2), and there was a significant difference in alkaloid composition between species within each site (Tarapoto, San Martin: Global R = 0.504, p = 0.016; Pongo de Cainarachi, San Martin: Global R = 0.424, p = 0.016; Sauce, San Martin: Global R = 0.76; p = 0.008; Varadero, Loreto: Global R = 0.636; p = 0.008; Figure 2).

**Figure 3:**
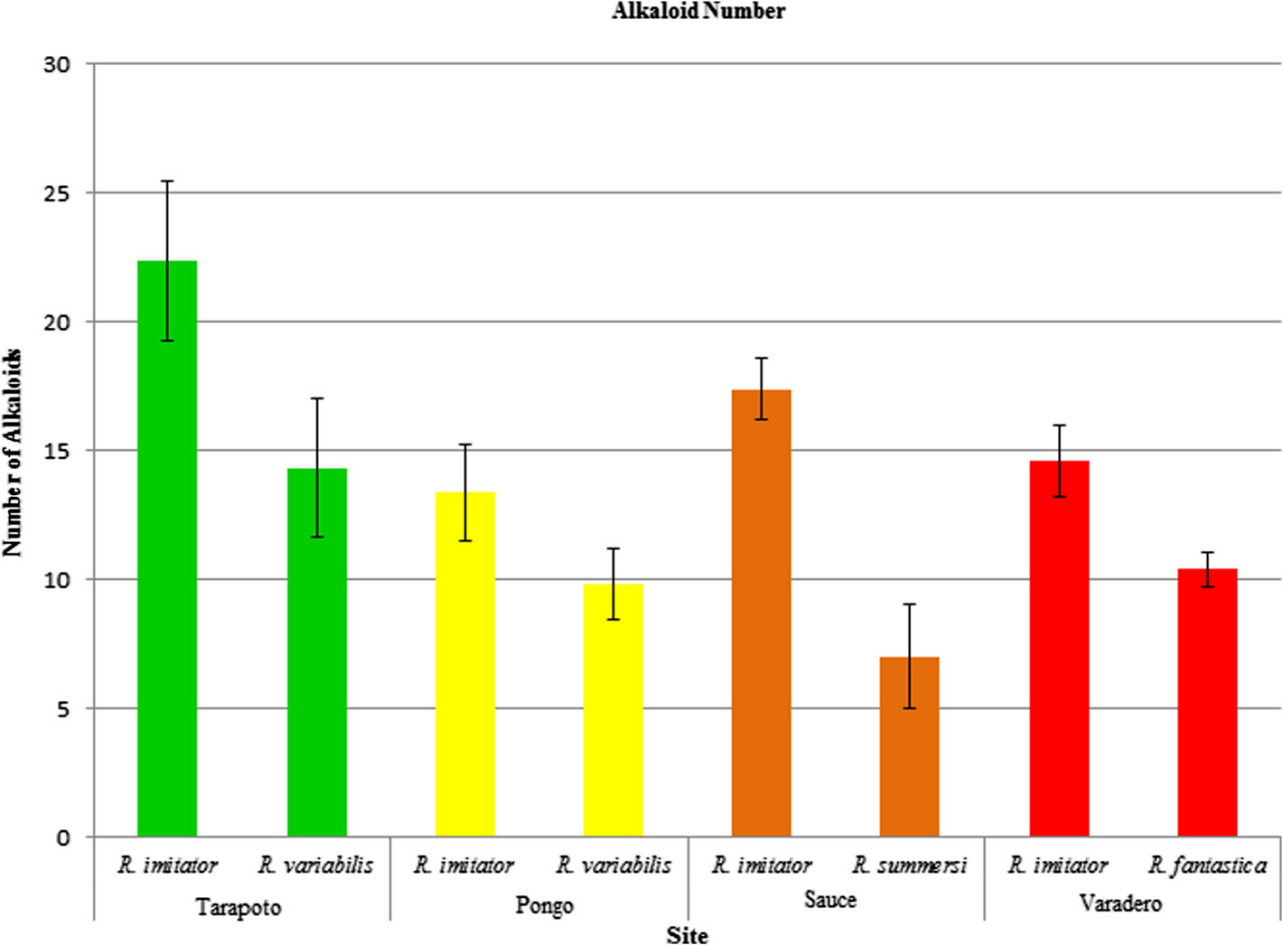
**Mean number of alkaloid types per species/site.** Error bars represent standard error of the mean

*Ranitomeya imitator* had a greater number of alkaloids than the co-existing mimetic species for each site. This difference was statistically significant in the Sauce banded morph of *R. imitator* and *R. summersi* (t_8_ = 4.451, p = 0.002) and the Varadero orange-headed morph of *R. imitator* and *R. fantastica* (t_8_ = 2.757, p = 0.025), and was suggestive of a trend in the Tarapoto spotted morph of *R. imitator* and *R. variabilis* (t_8_ = 1.857, p = 0.100) and the Pongo de Cainarachi striped population of *R. imitator* and *R. variabilis* (t_8_ = 1.549, p = 0.160; Figure 3).

**Figure 4:**
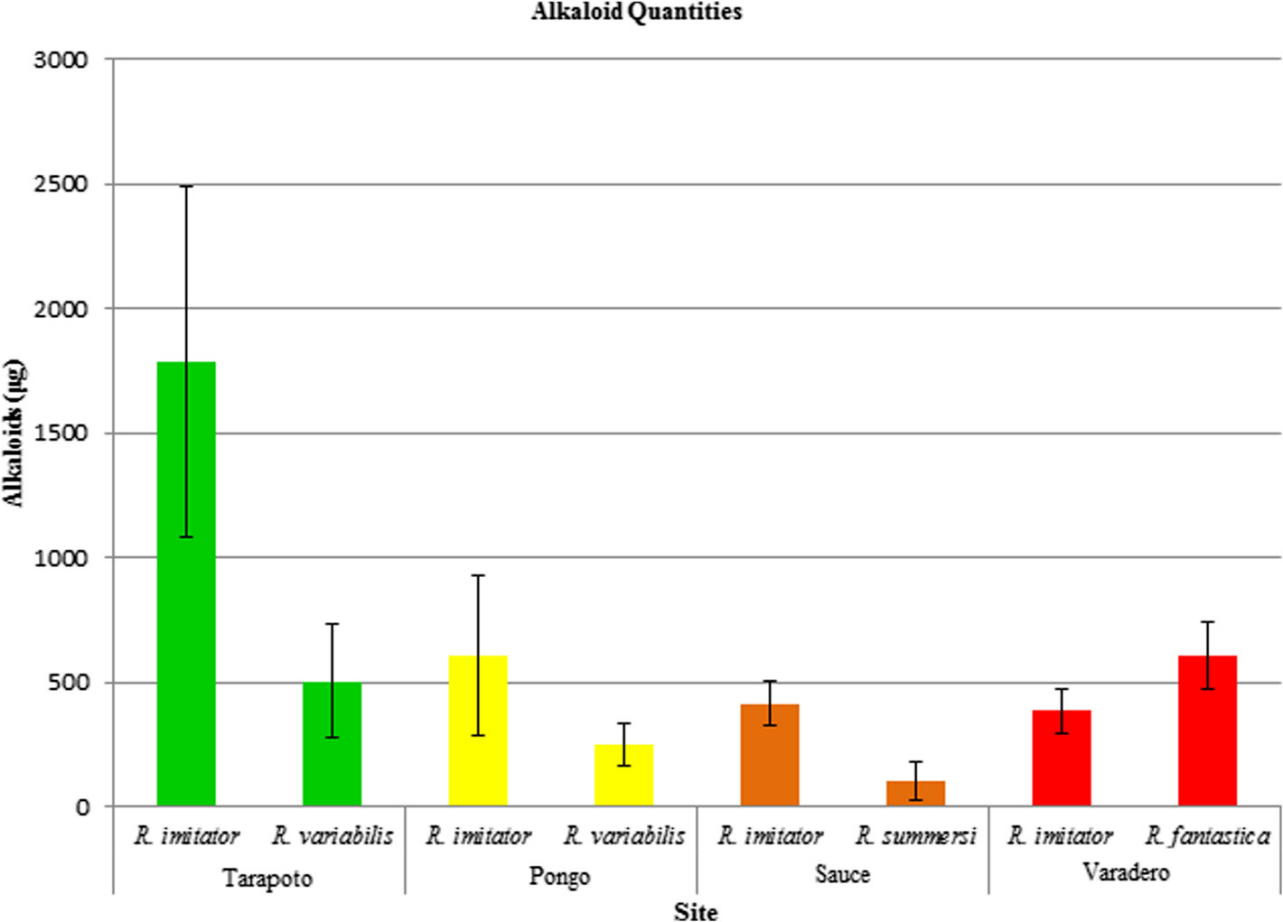
**Mean alkaloid quantities per species/site, corrected for frog mass.** Error bars represent standard error of the mean.

*Ranitomeya imitator* also had a greater quantity of alkaloids than its Müllerian co-mimic in every site except for the Varadero population (mimetic with *R. fantastica,* t_8_ = -1.355, p = 0.213). The variation in alkaloid quantity within mimetic species within a site is vast, however, and our sample size was low in an effort to reduce the number of frogs sacrificed. Thus, the difference is only statistically significant between *R. imitator* and *R. summersi* from Sauce (t_8_ = 2.671, p = 0.028) and the Tarapoto site with *R. imitator* and *R. variabilis* (t_8_ = 2.339, p = 0.047). The Pongo site (also *R. imitator* and *R. variabilis*) was not significant (t_8_ = 1.071, p = 0.315; Figure 4).

A total of 108 alkaloids, representing 13 different structural classes, were identified from skin extracts examined in this study. The most common and widespread alkaloids, found in at least one individual from each population, were the histrionicotoxins (HTX) **235A**, **259A**, **261A**, and **285A** (HTX **291A** was found in all populations except Tarapato, San Martin), the 2,5-disubstituted decahydroquinolines (DHQ) **219A** and **243A**, and the 3,5-disubstituted indolizidine (3,5-I) **223AB**. All of these alkaloids are likely derived from dietary ants [13,10], suggesting that ants play a major role in the chemical defenses of these frog populations. Data on the class, type, and quantity of alkaloids present within individual frogs are available in Additional file 1.

## Discussion

Numerous authors have postulated that the mimetic complexes of *Ranitomeya* represent a Müllerian system (e.g., [12,15,22-24]), and recent experimental evidence supports the hypothesis of Müllerian mimicry based on learned avoidance by predators [17,29]. One key prediction of Müllerian mimicry is the presence of chemical defenses in all mimetic species. Our data demonstrate for the first time that all *Ranitomeya* species involved in the mimetic complexes possess alkaloid defenses. This is a key tenet of Müllerian mimicry, and chemically defended co-mimetic species are likely to contribute to learned avoidance by predators. These findings provide further support of the hypothesis of Müllerian mimicry and evidence that all of these allopatric populations are likely an example of Müllerian mimicry.

Prior work examining mimicry among these mimetic species has posited that *R. variabilis* is less palatable to predators when compared to *R. imitator*[29]. Interestingly, the alkaloid data presented here illustrate that *R. imitator* contains significantly more alkaloids than its model species within most localities, suggesting that differences in palatability may not always be related to the number or quantity of alkaloids, but rather the specific types of alkaloids present in a frog. The manner in which individual alkaloids affect potential predators are virtually unknown, and similarly no data exist on the effects of complex alkaloid mixtures on potential predators. It is likely that different alkaloids possess different levels of palatability, and therefore differences in the presence or quantity of these particular alkaloids are most important to predator avoidance. Stuckert *et al.*[29] found no evidence that *R. imitator* elicited a greater avoidance response than the co-mimetic *R. variabilis*, suggesting that not all the differences in alkaloids are strongly correlated with predator response and, importantly, learned avoidance by predators.

There are two possible mechanisms to explain the difference in alkaloids between *R. imitator* and its congeners. The first is a genetic difference that might allow for more rapid or efficient sequestration in *R. imitator* when compared to *R. variabilis*. Although an intriguing possibility, this explanation seems improbable due to the close phylogenetic relationships between these species [11,12]. It is, however, possible that genes related to alkaloid sequestration are under a strong selective force and are rapidly evolving. The second, and more likely, explanation is a difference in arthropod diet, primarily resulting from differential microhabitat usage. Although these co-mimetic species frequently co-occur in the same habitats, *Ranitomeya imitator* is more commonly found in early secondary forests or disturbed portions of forest, due to the abundance of reproductive resources (e.g., *Heliconia* and *Diffenbachia sp*), whereas the co-mimetic model species (*R. fantastica, R. summersi,* and *R. variabilis*) are typically found in more pristine forest (AMMS pers. obs.) and are more arboreal [34]. The difference in microhabitat usage between these species may put *R. imitator* in contact with increased quantities of ant-derived toxins (e.g., DHQs, HTXs, and 3,5-Is) more frequently than its co-mimetic species. This is similar to *Mantella baroni,* which has been shown to possess more alkaloids in disturbed habitats [35]. Indeed, the data presented here indicate a significant difference in alkaloid composition between *R. imitator* and its model species, with much of this being driven by differences in quantity of the structurally similar ant-derived alkaloids. A detailed analysis of frog diet and arthropod abundance/distribution in this system would provide further insight on the observed differences in alkaloid defenses between species in this study.

Another possibility is that an increase in abundance of *R. imitator* compared to its initial mimetic radiation has decreased the selective pressure on its co-mimetic model species. *Ranitomeya imitator* adverged on to the appearance of already established congeneric species (Symula *et al.*[22,23]; however see [26] and above for discussion of this hypothesis), and presumably, *R. imitator* was under selective pressure to mimic already established species during this mimetic radiation [25]. However, since becoming established, *R. imitator* has become more abundant than its sympatric model species [25], thus we expect predators to come in to contact with *R. imitator* more frequently than the co-mimetic model species. As a result, the majority of predator learned avoidance will be driven by *R. imitator,* likely benefitting the rarer co-mimetic species [36]. If alkaloids are in fact costly to sequester [37,38], and dietary specialization is associated with increased metabolic rates [39], we might expect a reduction in alkaloid defenses of the co-mimetic species. Although speculative, the proliferation of *R. imitator* (and the associated increase of the frequency dependent mimetic signal) could reduce the pressure on these less common co-mimetic species to maintain high levels of alkaloid defense, thus allowing them to allocate resources away from sequestering alkaloids and towards reaching sexual maturity and reproduction.

Intriguingly, the Varadero locality where *R. imitator* is co-mimetic with *R. fantastica* is the only population in which the model species possessed a slightly greater quantity of alkaloids than *R. imitator* (although this difference was not statistically significant). *Ranitomeya fantastica* is present throughout much of the range of *R. imitator* included in this study (e.g., the Tarapoto and Pongo de Cainarachi populations), yet *R. imitator* evolved to mimic *R. variabilis* in these areas. Near Pongo de Cainarachi there is a population in which *R. imitator, R. variabilis,* and *R. fantastica* have all evolved a striped morph. However, this tri-mimicry occurs over a very restricted range, and *R. fantastica* is highly polytypic throughout the rest of the range of the striped morph of *R. variabilis* and *R. imitator.* As a result, we think that *R. variabilis* is the species that drove the evolution of this tri-mimicry*.* Given our alkaloid data, it remains unclear why *R. imitator* adverged on to *R. variabilis* throughout most of its range as opposed to *R. fantastica,* which possesses more alkaloids than *R. imitator* in the sympatric site we sampled (although this may not correlate with toxicity per se, see Discussion above). We propose that this occurred due to the greater phenotypic variation found in *R. fantastica* throughout this range, and because *R. variabilis* is more commonly encountered than *R. fantastica* in these areas [25], although it is possible that *R. imitator* looked more similar to the local *R. variabilis* than *R. fantastica* when they initially came in to contact. Aposematism and mimicry are frequency-dependent [15], thus we would expect a more abundant (or more commonly encountered species) to be a better model for an advergent mimic. If *R. imitator* evolved to mimic *R. variabilis* instead of *R. fantastica* due to abundance, this may indicate that differences in encounter rates can significantly influence the evolution of Müllerian mimicry, as predicted by theory.

Our data also indicate substantial intrapopulation variation in the alkaloid defenses between individuals (see nMDS plot in Figure 2). Similar results have been found in other poison frogs [1,3,19]. It is possible that automimicry in these mimetic complexes, particularly within *R. imitator,* may play a major role in both educating predators and maintaining learned avoidance in predators. Automimicry describes the existence of non-defended prey in sympatry with defended conspecifics [40,41], but the effects may be similar for species in which individuals vary greatly in their chemical defenses. Poison frogs sequester alkaloids from dietary sources [3] and the accumulation of these toxins likely results in automimicry within poison frog systems [19,42]. Automimicry is perhaps unsurprising in poison frog systems given that arthropod systems also exhibit substantial variation in toxicity and are automimetic (reviewed in [43]). Variation in alkaloid defenses within populations of poison frogs may result from the additive effect of temporal sequestration throughout life and patchy prey availability. Automimicry may be an important avenue of future research and we encourage theoretical studies of co-mimetic species that add a component of automimicry to the models.

## Conclusions

This study presents the most complete examination of alkaloid defense in the genus *Ranitomeya* to date, including analyses of four allopatric mimetic complexes of congenerics. This study demonstrates that all species from these allopatric mimetic complexes possess alkaloids, which is a key tenet of the hypothesis of Müllerian mimicry. Thus, these data provide further support of the hypothesis of Müllerian mimicry in these allopatric complexes. Coupled with prior data indicating reciprocal learned avoidance by predators in this system [17,29] this provides very strong evidence that these mimetic complexes are Müllerian in nature. We further provide evidence that raw alkaloid data may not correlate well with unpalatability (i.e., avoidance) from a predator’s perspective and additionally propose that automimicry may be acting in this system due to high levels of intrapopulation variation in alkaloid profiles.

## Methods

Specimens were collected from four sites within the departments of San Martin and Loreto, Peru during January and February 2012 (see Figure 1). These collections included 5 *R. imitator* and 5 *R. variabilis* from near Tarapoto, San Martin (18 January); 5 *R. imitator* and 5 *R. variabilis* from Pongo de Cainarachi, San Martin (12–15 January); 5 *R. summersi* and 5 *R. imitator* from Sauce, San Martin (24–27 January); and 5 *R. imitator* and 5 *R. fantastica* from Varadero, Loreto (8–10 February). After collection, frogs were euthanized and skins were placed in 4 mL, Teflon-lined glass vials filled with 100% methanol. Specimens were placed in the CORBIDI Herpetological Collection, Lima, Peru. Research permits were obtained through DGFFS in Lima, Peru (Resolución Directoral N° 033-2011-AG-DGFFS-DGEFFS) and our study protocol was approved by East Carolina University’s Institutional Animal Use and Care Committee (permit #D225). All work presented herein complied with the guidelines set forth by these governing agencies. Export permits are CARTA N° 1312–2011 - AG - DGFFS – DGEFFS.

Individual alkaloid fractions were prepared from methanol extracts of individual skin. For each sample 10 *μ*g of nicotine ((-)-nicotine ≥99%, Sigma-Aldrich, Milwaukee, Wisconsin) in a methanol solution (internal standard) and 50 *μ*L of 1 N HCl was added to 1 mL of the original MeOH extract. The combined MeOH extract was then concentrated with N_2_ to 100 *μ*L and then diluted with 200 *μ*L of water. The solution was extracted 4 times, each time with 300 *μ*L of hexane. The HCl fraction was then basified with saturated NaHCO_3_, followed by extraction 3 times, each time with 300 *μ*L of ethyl acetate. The combined ethyl acetate fractions were then dried with anhydrous Na_2_SO_4_ and evaporated to 100 *μ*L.

Gas chromatography–mass spectrometry (GC-MS) analysis was performed on a Varian Saturn 2100 T ion trap MS instrument coupled to a Varian 3900 GC with a 30 m × 0.25 mm i.d. Varian Factor Four VF-5 ms fused silica column. GC separation of alkaloids was achieved using a temperature program from 100 to 280°C at a rate of 10°C per minute with He as the carrier gas (1 mL/min). Each alkaloid fraction was analyzed with both electron impact MS and chemical ionization MS with methanol as the reagent gas.

Individual alkaloids were identified by comparing the observed MS properties and GC retention times with those of previously reported anuran alkaloids [44]. Anuran alkaloids have been assigned code names that consist of a bold-faced number corresponding to the nominal mass and a bold-faced letter to distinguish alkaloids of the same nominal mass [44]. To determine the quantity of alkaloids in frog skins, observed alkaloid peak areas were compared to the peak area of the nicotine internal standard, using a Varian MS Workstation v.6.9 SPI. Trace alkaloid peaks under 0.5 μg were excluded from our analyses.

The number and quantity of alkaloids were compared among species within a sampling site as well as between sites. The quantity of alkaloids per individual frog was corrected for frog mass and statistical tests use these corrected quantities for examining the alkaloid quantities unless otherwise noted. Independent samples t-tests were performed to compare the number and quantity of alkaloids between species within a sampling location. These statistical analyses were performed in SPSS v. 19. Non-metric multidimensional scaling (nMDS) was used to graphically visualize patterns of alkaloid composition (a combined measure of the type, number, and quantity of alkaloids) in frogs within and among sites. Analysis of similarity (ANOSIM) was used to detect differences in alkaloid composition among sites and between species within a site. All nMDS and ANOSIM analyses are based on Bray-Curtis dissimilarity matrices, and were performed using PRIMER-E version 5.

## Competing interests

We hereby confirm that we have no conflicts of interest.

## Authors’ contributions

AS participated in project design, conducted field work, and participated in lab work, data analysis, and manuscript writing. RS participated in project design, lab work, data analysis, and manuscript writing. PV participated in project design and manuscript writing. KS participated in project design and manuscript writing. All authors have read and approved the final manuscript.

## Supplementary Material

Additional file 1 Alkaloid data. Click here for file
