# Supplementary material for: Alkaloid defenses of co-mimics in a putative Müllerian mimetic radiation
Source: BMC Evol Biol. 2014 Apr 4;14:76. doi: 10.1186/1471-2148-14-76 (PMC4101839; doi:10.1186/1471-2148-14-76)
Supplement: Additional file 1 — Alkaloid data. [file 1471-2148-14-76-S1.pdf]

Appendix 1. Occurrence of alkaloids in skin extracts of individual *Ranitomeya imitator* , *Ranitomeya variabilis* , *Ranitomeya fantastica* , and *Ranitomeya summersii* arranged by mimetic location. Alkaloid quantities are as follows: **major alkaloids** (> 50 µg per skin), **minor alkaloids** (5-50 µg per skin), and **trace alkaloids** (< 5 µg per skin). The number in parentheses following an entry indicates the number of isomers.

**Location**<br>Species	**Frog**	**Alkaloid Quantity**	**Alkaloids**<br>**Ant origin:** HTX; DHQ; N-Me-DHQ; 4,6-Q; 3,5-I.<br>**Mite origin:** PTX; DesMe PTX; 5,8-I; 5,6,8-I; 1,4-Q; Unclass.<br>**Unnown origin:** OHQ; Izidine.
**Tarapoto, San Martin**<br>*Ranitomeya imitator*	1	Major	HTX **259A**, **261A**, **283A**, **285A**; DHQ **243A**(4).
		Minor	HTX **235A**; DHQ **219A**(2), **267L**; N-MeDHQ **237U**; 4,6-Q **195C**(2); 3,5-I 223AB(3); DesMe PTX **249G**.
		Trace	DHQ **249E**; 5,8-I **219J**; 5,6,8-I **231B**.
	2	Major	HTX **235A**(3); DHQ **219A**(5), **243A**(5).
		Minor	HTX **285A**(2), **287A**; DHQ **221D**(2), **269AB**.
		Trace	HTX **259A**, **261A**, **283A**(2); DHQ **249E**(2); 3,5-I **223AB**(3); Unclass **231I**.
	3	Major	HTX **235A**(3); DHQ **219A**(3).
		Minor	DHQ **221D**(2).
		Trace	5,8-I **219J**; Unclass **231I**.
	4	Major	DHQ **219A**(4), **243A**(7).
		Minor	HTX **235A**(2), **259A**; DHQ **223F**(2); 3,5-I **223AB**(3); Unclass **231I**.
		Trace	Unclass **209G**.
	5	Major	DHQ **219A**(3), **243A**(6).
		Minor	HTX **235A**(3); DHQ **223F**; 3,5-I **223AB**(3).
		Trace	HTX **259A**; 4,6-Q **195C**(2); Unclass **231I**.
**Tarapoto, San Martin**<br>*Ranitomeya variabilis*	1	Major	HTX **235A**(2).
		Minor	5,6,8-I **239Z**(2).
		Trace	HTX **283A**, **285A**; DHQ **219A**, **223F**.
	2	Major	
		Minor	
		Trace	HTX **235A**; DHQ **223F**, **243A**.

	3	Major	
		Minor	HTX **259A**, **261A**, **285A**; PTX **267A**; 5,6,8-I **231B**.
		Trace	HTX **235A**(2), **283A**; DHQ **221B**, **223F**, **251A**; 4,6-Q **195C**; PTX **251D**; 5,6,8-I **239Z**.
	4	Major	HTX **235A**(2); PTX **267A**.
		Minor	HTX **259A**, **261A**(2), **283A**(3), **285A**(2); DHQ **223F**; PTX **251D**; 5,6,8-I **231B**, **239Z**(2).
		Trace	DHQ **243A**, **251A**; 4,6-Q **195C**; PTX **237A**.
	5	Major	
		Minor	HTX **259A**, **285A**(2); DHQ **221B**, **223F**, **243A**, **251A**; 4,6-Q **195C**; PTX **267A**; 5,6,8-I **231B**.
		Trace	HTX **235A**, **261A**, **283A**; PTX **251D**; 5,6,8-I **239Z**.

**Pongo de Cainarachi,**	1	Major	HTX **235A**; DHQ **243A**(3).
**San Martin**		Minor	HTX **259A**, **261A**(2), **285A**, **291A**; DHQ **219A**(2), **223Q**.
Ranitomeya imitator		Trace	DHQ **219A**; 3,5-I **253T**; 5,6,8-I **237C**.
	2	Major	DHQ **219A**(4); **243A**(2).
		Minor	HTX **235A**, **259A**, **261A**(2), **285A**; DHQ **223Q**(2); 3,5-I **223AB**(2).
		Trace	5,6,8-I **223A**, **237C**, **239Z**.
	3	Major	
		Minor	HTX **259A**; DHQ **223Q**, **243A**(2).
		Trace	HTX **235A**, **261A**(2), **285A**, **291A**; DHQ **219A**; 5,6,8-I **223A**.
	4	Major	
		Minor	HTX **259A**, **291A**; DHQ **243A**(2).
		Trace	HTX **235A**, **261A**, **285A**; DHQ **219A**, **223Q**; 3,5-I **223AB**(2).
	5	Major	
		Minor	HTX **259A**, **291A**; DHQ **223Q**(2), **243A**(2).
		Trace	HTX **235A**, **261A**(2), **285A**.
**Pongo de Cainarachi,**	1	Major	
**San Martin**		Minor	DHQ **219A**(2), **223Q**; 3,5-I **181A**, **223AB**(3), **253T**(2).

Ranitomeya imitator		Trace	HTX **235A**, **259A**; DHQ **249E**, **253T**.
	2	Major	
		Minor	HTX **235A**, **259A**, **261A**.
		Trace	HTX **285A**, **291A**; DHQ **219A**, **243A**; 3,5-I **223AB**(2), **253T**.
	3	Major	
		Minor	DHQ **223Q**.
		Trace	HTX **259A**; DHQ **219A**, **243A**; 4,6-Q **195C**; 3,5-I **253T**; 5,6,8-I **231B**; 1,4-Q **231A**.
	4	Major	
		Minor	HTX **291A**; 3,5-I **253T**; 5,6,8-I **237C**.
		Trace	HTX **259A**, **261A**; DHQ **223Q**; 3,5-I **223AB**(3).
	5	Major	
		Minor	3,5-I **223AB**(3).
		Trace	3,5-I **253T**; 5,6,8-I **237C**.
**Sauce, San Martin**	1	Major	DHQ **243A**(3).
Ranitomeya imitator		Minor	HTX **259A**, **261A**, **285A**(2), **291A**; 3,5-I **223AB**(2); Unclass **209G**.
		Trace	DHQ **267L**, **269AB**; 5,8-I **243B**; 1,4-Q **257D**.
	2	Major	DHQ **243A**(3).
		Minor	3,5-I **223AB**(3).
		Trace	HTX **259A**, **261A**, **285A**, **291A**; DHQ **219A**, **267L**; 5,8-I **243B**; 1,4-Q **257D**; Unclass **209G**.
	3	Major	DHQ **243A**(4); 3,5-I **223AB**(3).
		Minor	HTX **291A**; DHQ **219A**, **269AB**; Unclass **209G**(2).
		Trace	HTX **259A**, **261A**, **285A**; DHQ **267L**; 4,6-Q **195G**; 5,8-I **243B**; 1,4-Q **257D**; Unclass **235P**, **231I**.
	4	Major	3,5-I **223AB**(3).
		Minor	HTX **291A**; DHQ **243A**(2), **269AB**; Unclass **209G**(2).
		Trace	HTX **259A**, **285A**; 4,6-Q **195G**; 5,8-I **243B**; 1,4-Q **257D**; Izidine **239X**(2); Unclass **235P**.
	5	Major	DHQ **219A**(3), **243A**(4).
		Minor	3,5-I **223AB**(3); Unclass **209G**.
		Trace	HTX **235A**, **259A**; DHQ **209J**, **267L**; 4,6-Q **195G**; 5,6,8-I **221P**; 1,4-Q **257D**; Unclass **231I**.

**Sauce, San Martin**	1	Major	
Ranitomeya summersii		Minor	
		Trace	HTX **283A**, **285A**; 3,5-I **223AB**; Unclass **209F**.
	2	Major	
		Minor	
		Trace	HTX **283A**, **285A**(2); 3,5-I **223AB**(2); Unclass **209F**.
	3	Major	
		Minor	HTX **283A**, **285A**(2); 3,5-I **223AB**(2).
		Trace	
	4	Major	
		Minor	DHQ **193D**.
		Trace	HTX **283A**, **285A**; 3,5-I **223AB**(2).
	5	Major	HTX **259A**, **283A**, **285A**.
		Minor	HTX **235A**; DHQ **243A**(2).
		Trace	HTX **261A**(2); N-Me-DHQ **237U**; 3,5-I **223AB**(2); PTX **323A**; 5,8-I **243B**; 1,4-Q **257D**; OHQ **193D**.
**Varadero, Loreto**	1	Major	DHQ **219A**(2).
Ranitomeya imitator		Minor	DHQ **243A**(3); 5,6,8-I **263D**.
		Trace	HTX **235A**, **259A**, **291A**; Unclass **209G**, **231I**.
	2	Major	DHQ **219A**(3).
		Minor	HTX **235A**, **259A**, **261A**, **285A**, **291A**; DHQ **243A**(3); Unclass **209G**.
		Trace	DHQ **223F**; 5,6,8-I **263D**; Unclass **231I**.
	3	Major	DHQ **219A**(2).
		Minor	HTX **235A**, **291A**; N-Me-DHQ **237U**(2).
		Trace	HTX **259A**, **281A**; DHQ **223F**, **243A**(2); 4,6-Q **195C**; 3,5-I **223AB**(2); 5,6,8-I **263D**; Unclass **209F**, **231I**.
	4	Major	DHQ **219A**(3).
		Minor	HTX **235A**, **291A**; DHQ **243A**(3); 4,6-Q **195C**(2); Unclass **209F**, **231I**.
		Trace	HTX **259A**, **281A**; DHQ **211A**, **223F**; 3,5-I **223AB**; 5,6,8-I **263D**.

	5	Major	HTX **291A**; DHQ **219A**(2).
		Minor	HTX **235A**, **259A**; DHQ **243A**; 5,6,8-I **263D**.
		Trace	HTX **261A**, **281A**; Unclass **209F**, **231J**.
**Varadero, Loreto**	1	Major	HTX **291A**.
Ranitomeya fantastica		Minor	HTX **235A**(2), **259A**(2), **285A**; 5,6,8-I **263D**.
		Trace	HTX **261A**; DHQ **219A**, **223F**; 5,6,8-I **253H**.
	2	Major	HTX **291A**; DHQ **223F**(3).
		Minor	HTX **235A**.
		Trace	DHQ **219A**; 3,5-I **223AB**; 5,6,8-I **253H**.
	3	Major	HTX **259A**, **285A**, **291A**.
		Minor	HTX **235A**(2), **261A**.
		Trace	HTX **283A**; DHQ **219A**, **243A**; 5,6,8-I **253H**.
	4	Major	HTX **235A**(2), **291A**.
		Minor	DHQ **223F**(2).
		Trace	DHQ **219A**(2); 3,5-I **223AB**; 5,6,8-I **253H**; Unclass **209F**.
	5	Major	HTX **259A**, **285A**, **291A**; DHQ **243A**.
		Minor	HTX **235A**, **261A**(2).
		Trace	DHQ **219A**(2), **223F**, **269B**; 3,5-I **223AB**.

HTX (histrionicotoxin); DHQ (2,5-disubstituted decahydroquinoline); N-Me-DQH (N-methyl-decahydroquinoline); 4,6-Q (4,6-disubstituted quinolizidine); 3,5-I (3,5-disubstituted indolizidine); PTX (pumiliotoxin); DesMe PTX (desmethylpumiliotoxin); 5,8-I (5,8-disubstituted indolizidine); 5,6,8-I (5,6,8-trisubstituted indolizidine); 1,4-Q (1,4-disubstituted quinolizidine); OHQ (octahydroquinoline); Izidine (Unclassified Izidines); Unclass (Unclassified alkaloids).
